# New highly sensitive rodent and human tests for soluble amyloid precursor protein alpha quantification: preclinical and clinical applications in Alzheimer’s disease

**DOI:** 10.1186/1471-2202-13-84

**Published:** 2012-07-23

**Authors:** Christiane Rose, Katell Peoc’h, Stéphanie Chasseigneaux, Claire Paquet, Julien Dumurgier, Fanchon Bourasset, Frédéric Calon, Jean-Louis Laplanche, Jacques Hugon, Bernadette Allinquant

**Affiliations:** 1 INSERM UMR 894, Université Paris Descartes, Sorbonne Paris Cité, Faculté de Médecine, Paris, France; 2 Service de Biochimie et de Biologie moléculaire, Hôpital Lariboisière, AP-HP, Paris, France; Biologie cellulaire, Faculté de Pharmacie, Université Paris Descartes, Paris, France; 3 Centre Mémoire de Ressources et de Recherche Paris Nord Ile de France, Groupe Hospitalier Lariboisière Fernand Widal Saint-Louis, Université Paris VII, Paris, France; 4 Laboratoire d’Histologie et de Biologie du Vieillissement, Groupe Hospitalier Lariboisière Fernand Widal Saint-Louis, Université Paris VII, Paris, France; 5 INSERM U839, Institut du Fer à Moulin, Paris, France; 6 Faculty of Pharmacy, Laval University, Quebec, Canada

**Keywords:** Alzheimer’s disease, Soluble amyloid precursor protein alpha, Homogeneous time-resolved fluorescence, Rodent, Human, Cerebrospinal fluid, Primary neurons, Sensitivity

## Abstract

**Background:**

Amyloid precursor protein (APP), a key molecule in Alzheimer’s disease (AD), is metabolized in two alternative cleavages, generating either the amyloidogenic peptides involved in AD pathology or the soluble form of APP (sAPPα). The level of amyloidogenic peptides in human cerebrospinal fluid (CSF) is considered to be a biomarker of AD, whereas the level of sAPPα in CSF as a biomarker has not been clearly established. sAPPα has neurotrophic and neuroprotective properties. Stimulating its formation and secretion is a promising therapeutic target in AD research. To this end, very sensitive tests for preclinical and clinical research are required.

**Methods:**

The tests are based on homogenous time-resolved fluorescence and require no washing steps.

**Results:**

We describe two new rapid and sensitive tests for quantifying mouse and human sAPPα. These 20 μl-volume tests quantify the levels of: i) endogenous mouse sAPPα in the conditioned medium of mouse neuron primary cultures, as well as in the CSF of wild-type mice, ii) human sAPPα in the CSF of AD mouse models, and iii) human sAPPα in the CSF of AD and non-AD patients. These tests require only 5 μl of conditioned medium from 5 × 10^4^ mouse primary neurons, 1 μl of CSF from wild-type and transgenic mice, and 0.5 μl of human CSF.

**Conclusions:**

The high sensitivity of the mouse sAPPα test will allow high-throughput investigations of molecules capable of increasing the secretion of endogenous sAPPα in primary neurons, as well as the *in vivo* validation of molecules of interest through the quantification of sAPPα in the CSF of treated wild-type mice. Active molecules could then be tested in the AD mouse models by quantifying human sAPPα in the CSF through the progression of the disease. Finally, the human sAPPα test could strengthen the biological diagnosis of AD in large clinical investigations. Taken together, these new tests have a wide field of applications in preclinical and clinical studies.

## Background

Alzheimer’s disease (AD) is a neurodegenerative disease characterized by cognitive deficits leading to a progressive loss of functional autonomy. The neuropathological hallmarks of the disease are extracellular amyloid deposits and intracellular neurofibrillar tangles containing phosphorylated Tau (pTau) [1]. According to recently proposed AD diagnostic criteria, diagnosis is based on clinical criteria, medical history, neuropsychological evaluation, morphological neuroimaging, cerebrospinal (CSF) biomarkers and/or molecular imaging (PET scan) [2]. AD is biologically characterized by the combination of low Aβ1-42 peptide levels and high levels of total Tau and Tau phosphorylated on threonine 181 (pTau181) in the CSF [3-6]. However, new biomarkers are needed to better discriminate AD from non-AD patients and to improve the diagnosis of AD in patients with atypical CSF profiles.

Amyloid precursor protein (APP) is a key molecule in AD pathology. Proteolytic cleavages of APP by the sequential actions of β- and γ-secretases form the neurotoxic amyloid beta (Aβ) peptide, which typically consists of 40 or 42 amino acid residues (the amyloidogenic pathway). An alternative cleavage by α-secretase, which splits the APP sequence in the middle of the amyloid peptide sequence, prevents formation of the Aβ peptide and releases the resulting APP N-terminal ectodomain (sAPPα)[7-9]. The physiological regulation of β and α cleavages is poorly established. However, recent papers describe an α cleavage following the β cleavage, as the C-terminal fragment (C99) released after β-secretase cleavage can be processed in α-secretase to generate the C83 fragment [10]. In addition, truncated Aβ peptide of 14 to 16 residues generated from the sequential actions of β and α-secretases have been identified in the CSF [11]. Interestingly, an inhibitory domain for γ-secretase activity in the C83 fragment has been identified able to down regulate the production of Aβ peptide [12]. In addition to its regulation of Aβ peptide, α-secretase cleavage appears very important as sAPPα is involved in several functions [13,14]. sAPPα is known for its neurotrophic and neuroprotective properties [13,14]. In addition, it stimulates the proliferation of neuroblasts from the sub-ventricular zone and increases long-term potentiation [15,16].

Recent studies have investigated sAPPα as a putative biomarker of AD [17,18] and tested the hypothesis that dysregulation of the alternative cleavage of APP may contribute to the pathophysiology of the disease. In the past, human sAPPα has been evaluated by western blotting [19-22], ELISA [23,24], and multiplex technology [17,18,25]. However, some of the findings regarding sAPPα levels in human CSF have been contradictory. Such discordant results signal the need for an ideal test that allows very sensitive and specific detection of sAPPα in small amounts of human CSF. Recently, a new technology (Alpha Screen^TM^) [26] requiring no washing and using low sample volume has been applied to sAPPα (AlphaLISA) but no results have been published yet.

From a research perspective, sAPPα quantification in preclinical studies in rodents is also necessary to better understand AD pathophysiology and to develop new therapeutic targets. Different cell lines, which include neuroblastoma cells, are currently being used to study APP metabolism *in vitro*. Although rodent primary neurons produce low amounts of Aβ peptide [27], this model remains interesting to study the response of sAPPα to AD therapeutic drug candidates before their *in vivo* validation in mice or rats. To effectively evaluate these models, a sensitive, reproducible and rapid test is needed to accurately assess sAPPα levels in the conditioned medium of rodent neuron primary cultures and to create high-throughput drug screening assays. Tests are also needed for the *in vivo* evaluation of candidate drugs identified in *in vitro* screens, both in wild-type mice and in transgenic AD mouse models.

We describe two new rapid and sensitive sAPPα tests that are based on homogenous time-resolved fluorescence (HTRF) and can be used to quantify the levels of: i) endogenous rodent sAPPα in the conditioned medium of mouse primary neurons, as well as in the CSF of wild type mice; ii) human sAPPα in the CSF of AD mouse models; and iii) human sAPPα in the CSF of AD and non-AD patients. These tests require only 5 μl of conditioned medium from 5 × 10^4^ mouse primary neurons, 1 μl of CSF from wild-type and transgenic AD mouse models or 0.5 μl of human CSF.

## Results and discussion

**Figure 1:**
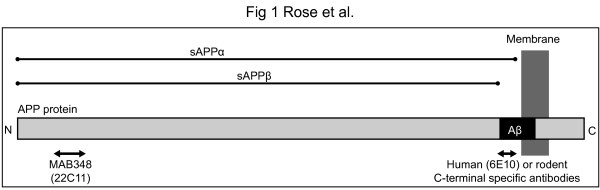
**Antibodies used for human and rodent sAPPα tests.** For the human assay, the antibody 6E10 (specific for h-Aβ1-16) binds to a h-sAPPα epitope, which differs from the r-sAPPα by three amino acids. In the rodent assay, this antibody is substituted by a rodent Aβ1-16-specific antibody. Furthermore, this epitope is absent from sAPPβ, hence, these two C-terminal sAPPα antibodies determine the specificity of the assay whereas MAB 348 (22C11) detects both human and rodent N-terminal fragments.

We developed two new sAPPα assays using an adaptation of the HTRF technology: one detects mouse/rodent-sAPPα (r-sAPPα) and the other human-sAPPα (h-sAPPα) through the application of two different APP antibodies (Figure 1). Both tests are single-step homogenous assays that require no washing unlike most ELISA procedures. This absence of multiple washing steps increases the speed and reproducibility of the assay. The final volume of the test is 20 μl, which allows detection in a 384-well plate for high-throughput investigations. These tests are extremely easy to use and require few preanalytical preparations.

### Analytical performance of the HTRF sAPPα assays

**Figure 2:**
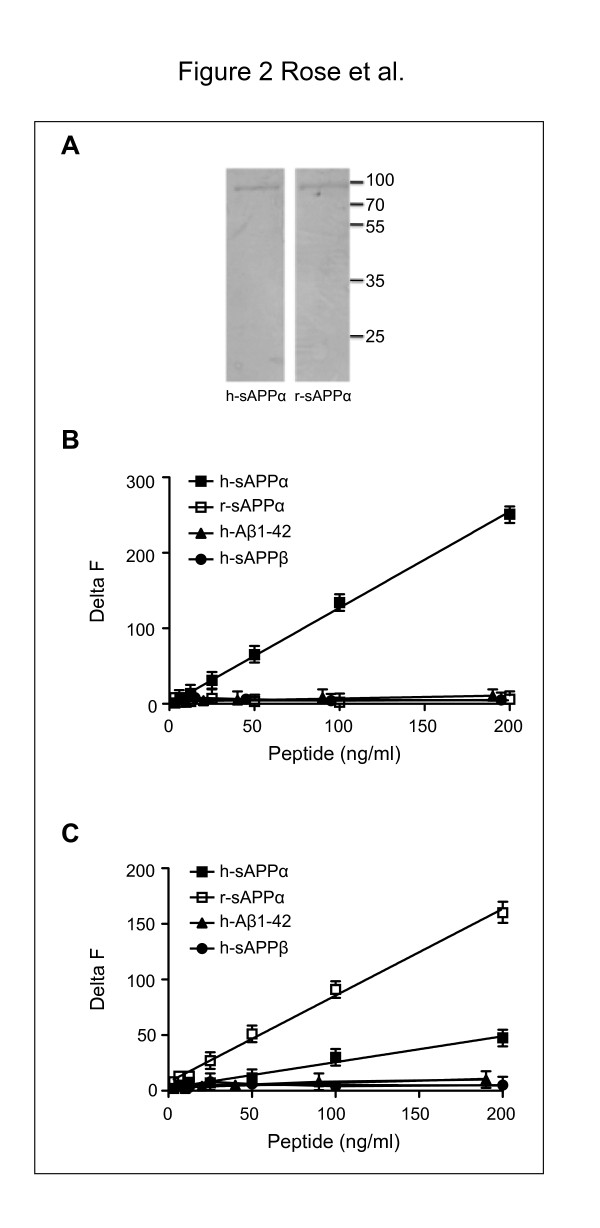
**Specificity of the assays.A**: Recombinant proteins h-sAPPα and r-sAPPα purity. 0.5 μg of h-sAPPα and r-sAPPα separated in 10% SDS-PAGE and stained with Simply Blue Safe Stain. The purity of both recombinant proteins is shown by the absence of additional bands. **B**: Human sAPPα assay. Calibration curves were generated with various concentrations of the recombinant proteins h-sAPPα, h-sAPPβ, r-sAPPα and the commercially available human peptide h-Aβ1-42. Results were calculated from the 665 nm/620 nm ratios and expressed in delta F. Slope for h-sAPPα: 1.47 ± 0.05; slopes for other peptides: <0.05. **C**: Rodent sAPPα assay. Calibration curves were generated with various concentrations of the recombinant proteins r-sAPPα, h-sAPPβ, h-sAPPα and the commercially available peptide h-Aβ1-42. Results were calculated from the 665 nm/620 nm ratios and expressed in delta F. Slope for r-sAPPα: 0.73 ± 0.02; slope for h-sAPPα: 0.17 ± 0.01; slopes for other peptides: <0.05.

The purity of the standard recombinant proteins h-sAPPα and r-sAPPα was checked by SDS-PAGE (Figure 2A).We evaluated the specificity of the h-sAPPα assay using human-sAPPβ (h-sAPPβ) and r-sAPPα recombinant proteins, which both contain the MAB 348 epitope, and the human peptide Aβ1-42 (h-Aβ1-42), which contains the 6E10 epitope; both epitopes are necessary for h-sAPPα assay. The signal obtained with 3 − 200 ng/ml of h-sAPPβ, r-sAPPα and h-Aβ1-42 was similar to that obtained for the blank, demonstrating that the h-sAPPα does not cross-react with any of these negative controls (Figure 2B). We evaluated the specificity of the r-sAPPα assay using recombinant h-sAPPα, h-sAPPβ and the h-Aβ1-42 peptide. We detected a cross-reactivity level of 23% with h-sAPPα, indicating that the rodent C-terminal sAPPα antibody is less specific than the human sAPPα 6E10 antibody (slope for r-sAPPα: 0.73 ± 0.02, slope for h-sAPPα: 0.17 ± 0.01). No cross-reactivity was observed with h-sAPPβ or h-Aβ1-42 (Figure 2C).

**Figure 3:**
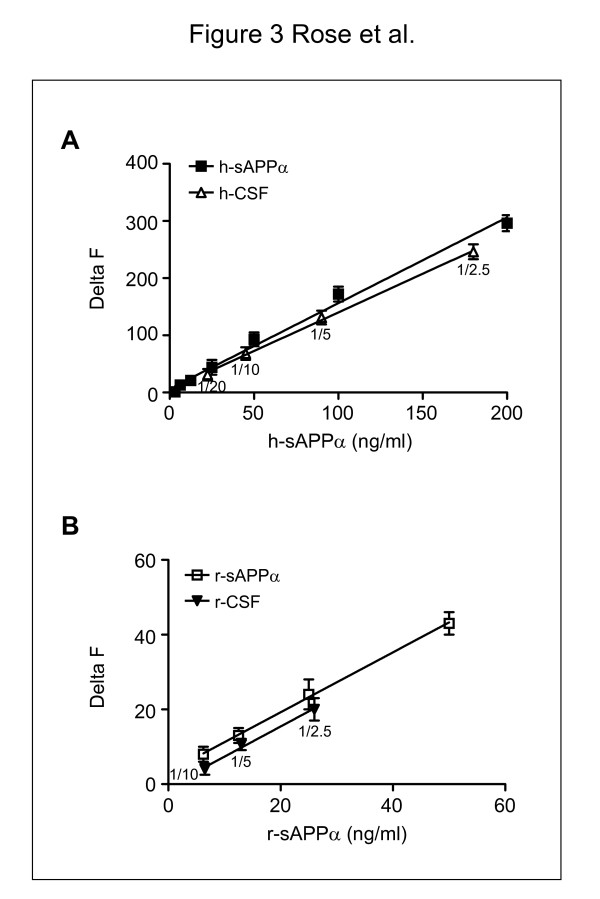
**Linearity of the CSF sAPPα assays**. **A**: Human sAPPα assay. Serial dilutions of h-CSF between 1/2.5 and 1/20 were assayed and compared to serial dilutions of recombinant h-sAPPα at concentrations between 0 ng/ml and 200 ng/ml. Results were calculated from the 665 nm/620 nm ratios and expressed in delta F. Slope for h-CSF: 1.35 ± 0.04; slope for recombinant h-sAPPα: 1.45 ± 0.05. **B**: Rodent sAPPα assay. Serial dilutions of r-CSF from 1/2.5 to 1/10 were assayed and compared to serial dilutions of recombinant r-sAPPα at concentrations between 0 ng/ml and 50 ng/ml. Results were calculated from the 665 nm/620 nm ratios and expressed in delta F. Slope for r-CSF: 0.83 ± 0.04; slope for recombinant r-sAPPα: 0.76 ± 0.04.

We obtained a linear response using 3 − 200 ng/ml of recombinant h-sAPPα or r-sAPPα, which indicated that the limit of detection was approximately 3 ng/ml (Figure 2B, C). We then evaluated the linearity of both assays with serial dilutions of human (h-) or mouse (r-) CSF. In both cases, the slopes obtained with h-CSF (1.39 ± 0.04) and r-CSF (0.90 ± 0.07) were parallel to the standard curves generated by recombinant human (1.49 ± 0.05) and rodent (0.88 ± 0.06) sAPPα, respectively. These results demonstrate that sAPPα levels in the CSF can be accurately evaluated in these assays with only 0.5 μl (5 μl diluted 1/10) of h-CSF or 1 μl (5 μl diluted 1/5) of r-CSF (Figure 3A, B).

To assess the intra- and inter-assay imprecision of the h-sAPPα assay, five h-CSF samples were each tested ten times on one plate in the same experiment and were tested three times in three independent experiments. The mean values for intra- and inter-assay imprecision were <5% with one exception in each experiment. Imprecision was 6.6% for one CSF sample in the intra-assay experiment and 8.3% for a different CSF sample in the inter-assay experiment (Table 1).

**Table 1:** Intra- and inter-assay imprecision in the h-sAPPα

**Intra-assay imprecision**		
**Human CSF sample**	**h-sAPPα (ng/ml)**	**CV (%)**
1	705 ± 13	1.8
2	432 ± 18	4.1
3	434 ± 16	3.6
4	556 ± 21	3.7
5	270 ± 18	6.6
**Inter-assay imprecision**		
**Human CSF sample**	**h-sAPPα (ng/ml)**	**CV (%)**
1	703 ± 24	3.4
2	424 ± 21	4.9
3	503 ± 17	3.3
4	493 ± 41	8.3
5	338 ± 17	5.0

Intra-assay imprecision: five CSF samples were tested ten times on one plate.

Inter-assay imprecision: the same samples were tested three times in three independent experiments. Mean ± SEM of h-sAPPα concentration (ng/ml). CV: coefficient of variation (%).

To assess the intra- and inter-assay imprecision of the r-sAPPα assay, three samples of conditioned media from mouse neuron primary cultures were each tested ten times on one plate in the same experiment and were each tested three times in three independent experiments. The mean values for the intra- and inter-assay imprecision were below 6% and 7%, respectively, with one exception of 11.4% for one sample in the inter-assay experiment (Table 2).

**Table 2:** Intra- and inter-assay imprecision in the r-sAPPα assay

**Intra-assay imprecision**		
**Neuron conditioned medium**	**r-sAPPα (ng/ml)**	**CV (%)**
1	23.4 ± 1.4	5.9
2	15.9 ± 0.7	4.4
3	17.4 ± 0.6	3.4
**Inter-assay imprecision**		
**Neuron conditioned medium**	**r-sAPPα (ng/ml)**	**CV (%)**
1	24.7 ± 1.5	6.0
2	14.8 ± 1.7	11.4
3	18.6 ± 1.2	6.4

Intra-assay imprecision: three conditioned media samples from three different experiments were tested ten times on one plate.

Inter-assay imprecision: the same samples were tested three times in three independent assays. Mean ± SEM of r-sAPPα concentration (ng/ml). CV: coefficient of variation (%).

We then examined the stability of h-CSF sAPPα. First, we evaluated the effect of storage in polypropylene or polystyrene tubes. Five samples were divided into polypropylene or polystyrene tubes and stored for 24 hours at −80°C. The h-sAPPα values of samples stored in polystyrene tubes were not significantly different from those of samples stored in polypropylene tubes (mean recovery: 93 ± 2%; Table 3), which is a real concern in a clinical practice [28-30].

**Table 3:** Stability of h-sAPPα in CSF stored at different conditions

**Effect of storing CSF in polypropylene and polystyrene tubes**			
**Samples**	**Polypropylene**	**Polystyrene**	**%**
	**h-sAPPα (ng/ml)**	**h-sAPPα (ng/ml)**	
1	669 ± 20	659 ± 41	98 ± 6
2	399 ± 20	391 ± 24	98 ± 6
3	456 ± 2.5	412 ± 16	90 ± 3
4	535 ± 16	482 ± 18	90 ± 3
5	236 ± 2	212 ± 7	89 ± 3

Five CSF samples were divided into polypropylene or polystyrene tubes and stored for 24 hours at −80°C. Mean ± SEM of h-sAPPα in ng/ml and (%) compared to the aliquot stored in polypropylene tubes.

Next, we evaluated whether storage temperature affected the stability of h-sAPPα. Five aliquots of h-CSF were stored for 24 hours at −80°C, −20°C, +4°C or room temperature, or were stored at −80°C and subjected to three freeze/thaw cycles during three days. No significant differences were detected for the different storage conditions or for the samples that underwent the three freeze/thaw cycles. Thus, sAPPα in CSF appears to be very stable (Table 4).

**Table 4:** Stability of h-sAPPα in CSF stored at different conditions

**Effect of storage temperature**					
**Samples**	**−80°C**	**−20°C**	**+4°C**	**RT**	**F/T**
1	100 ± 6	101 ± 5	100 ± 11	93 ± 1	103 ± 2
2	100 ± 7	93 ± 9	95 ± 15	85 ± 14	93 ± 6
3	100 ± 1	77 ± 1	82 ± 4	80 ± 4	76 ± 4
4	100 ± 5	113 ± 2	80 ± 4	109 ± 3	99 ± 5
5	100 ± 3	86 ± 5	81 ± 4	70 ± 5	83 ± 1

Five CSF samples were divided in five aliquots and stored for 24 hours at one of several different temperatures including room temperature (RT), or stored at −80°C and exposed to three freeze/thaw cycles (F/T) within a period of three days.

The results were normalized (%) for the results from the aliquot stored at −80°C.

### sAPPα assay development: *in vitro* applications

We used our sAPPα assays to measure the sAPPα levels in the conditioned media of human neuroblastoma cells and mouse neuron primary cultures.

**Figure 4:**
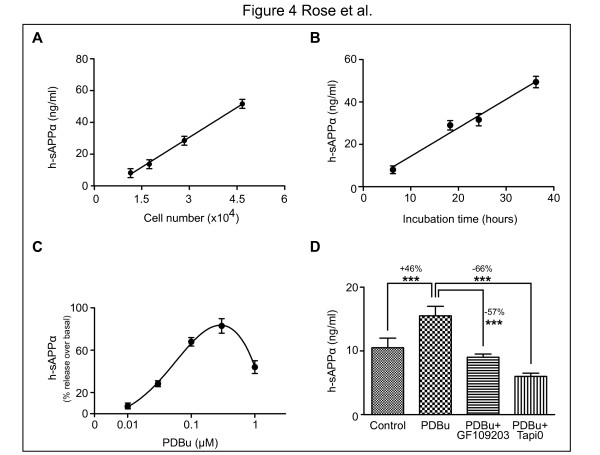
***In vitro*secretion of h-sAPPα by SH-SY5Y human neuroblastoma cells and its modulation by pharmacologic agents.A**: Various numbers of SH-SY5Y human neuroblastoma cells (between 10^4^ and 5 × 10^4^) were plated in 96-well plates in 0.1 ml final volume. After a 24 hours incubation, medium was collected and 5 μl of each sample was tested in duplicate in the h-sAPPα assay. **B**: 3 × 10^4^ SH-SY5Y cells were plated in 96-well plates in 0.1 ml final volume. Medium was collected after incubations of 6 hours, 18 hours, 24 hours or 36 hours, 5 μl of each sample was tested in duplicate in the h-sAPPα assay. **C**: 3 × 10^4^ SH-SY5Y cells were plated in 96-well plates in 0.1 ml final volume. The next day, the conditioned medium was discarded and replaced by the same medium containing 1% N2 in place of FCS. Various concentrations of PDBu (0.01 − 1 μM) were then added. After an incubation of 24 hours, medium was collected and 5 μl of each sample was tested in duplicate in the h-sAPPα assay. **D**: 3 × 10^4^ SH-SY5Y cells were plated in 96-well plates in 0.1 ml final volume. The next day, the conditioned medium was discarded and replaced by the same medium containing 1% N2 in place of FCS. Then, GF109203 (10 μM) and TAPI 0 (30 μM) were added and PDBu (0.3 μM) was added 1 hour later. After a 24 hour incubation, the medium was collected and 5 μl of each sample was tested in duplicate in the h-sAPPα assay. ***: p < 0.001.

Human neuroblastoma cells and mouse primary neurons were plated in 96-well plates. We first determined whether sAPPα secretion was proportional to the number of cells per well and to the time of incubation (Figure 4A, B). sAPPα secretion from cultured cells can be enhanced *in vitro* by the PKC-mediated stimulation of the metalloprotease ADAM17 [31-33]. The neuropeptide PACAP 27 [34] promotes the α-secretase-mediated cleavage of full-length APP through the constitutive ADAM10 metalloprotease. The green tea polyphenol EGCG stimulates either ADAM10 or ADAM17, depending on the cell type being treated [35,36]. We treated 3 x 10^4^ neuroblastoma cells with PDBu (a PKC activator) to evaluate its impact on the levels of sAPPα produced in conditioned media. We demonstrated that 0.01 to 0.3 μM of PDBu treatment caused a dose-dependent increase in the level of h-sAPPα in the conditioned medium of neuroblastoma cells (Figure 4C). This increase was completely abolished by treatment with the PKC inhibitor GF-109203 or the α-secretase inhibitor TAPI 0 (Figure 4D).

**Figure 5:**
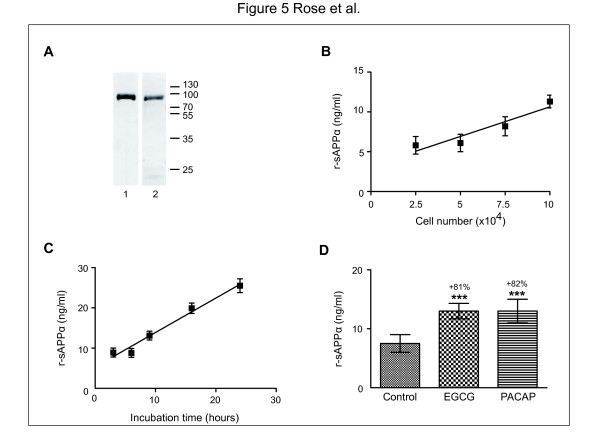
***In vitro*secretion of r-sAPPα by cortical neurons in primary culture and its modulation by pharmacologic agents.A**: The conditioned medium of 4 x 10^5^ neurons at six days *in vitro* was precipitated by a mixture of trichloraceticacid/acetone and the pellet was resupended in Laemmli buffer. An equal volume was loaded in two wells in 10% SDS-PAGE for western blotting. r-sAPPα at 100 kDa was recognized both by 22C11 (2) and by the rodent C-terminal sAPPα antibodies (1). **B**: Various numbers of cortical neurons (between 10^4^ and 7 × 10^4^) were plated in 96-well plates in 0.1 ml final volume. After six days in culture, 60% of the conditioned medium was discarded and replaced by fresh medium. 24 hours later, medium was collected and 5 μl of each sample was tested in duplicate in the r-sAPPα assay. **C**: 5 × 10^4^ neurons were plated in 96-well plates in 0.1 ml final volume. After six days *in vitro*, 60% of the conditioned medium was discarded and replaced by fresh medium. Medium was collected after incubations of 3 hours, 6 hours, 9 hours or 24 hours and 5 μl of each sample was tested in duplicate in the r-sAPPα assay. **D**: 5 × 10^4^ neurons were plated in 96-well plates in 0.1 ml final volume. After six days *in vitro*, 60% of the conditioned medium was discarded and replaced by fresh medium. EGCG (30 μM) and PACAP-27 (1 μM) were added and the medium was collected 24 hours later. 5 μl of each sample was tested in duplicate in the r-sAPPα assay. ***: p < 0.001.

To further validate the new r-sAPPα assay, we used mouse primary neurons, at six days *in vitro*. We first checked by western blotting the presence of r-sAPPα in the conditioned medium recognized by both 22C11 and rodent specific C-terminal antibodies (Figure 5A). This sAPPα band at 100 kDa is specific of the isoform 695 present in neurons [37].

Mouse primary neurons were plated in 96 well-plates at the density of 5 × 10^4^ cells per well. We checked that r-sAPPα secreted was proportional to the number of neurons and to the time of incubation (Figure 5B,C). Treatment of primary neurons with 30 μM EGCG or with 1 μM PACAP-27 produced a strong increase in the level of endogenous r-sAPPα in the incubation medium (Figure 5D). These results from our rodent test validate previously reported western blotting data from treated neuroblastoma cells [34,35].

The rodent test detected sAPPα in only 5 μl of conditioned medium from 5 × 10^4^ primary neurons cultured in 96-well plates, without concentration of the proteins. Five CSF samples were divided into polypropylene or polystyrene tubes and stored for 24 hours at −80°C. Mean ± SEM of h-sAPPα in ng/ml and (%) compared to the aliquot stored in polypropylene tubes. In previously published reports, evaluating sAPPα in conditioned media required higher numbers of cells combined with trichloracetic acid precipitation of proteins [34], media lyophilization [35] or protein concentration with centrifugal filters [36].

### sAPPα assay development: *in vivo* applications in mice

To evaluate the feasibility of measuring sAPPα levels in the CSF of mice, we used 14-month-old wild-type mice and 12-month-old AD triple transgenic mice (3xTg AD) [38]. The maximum volume of CSF obtained from one mouse was 5 μl. sAPPα assays were performed on each CSF sample diluted 1/5 or 1/10. The mean r-sAPPα concentrations in the CSF were 137 ± 17 ng/ml for wild-type mice and 105 ± 8 ng/ml for 3xTg AD mice. In the 3×Tg AD mice, the h-sAPPα concentration was 34 ± 6 ng/ml (Table 5). In the 3×Tg AD mice, the h-sAPPα derived from the transgene was specifically and distinctly quantified in the CSF relative to the endogenous r-sAPPα, whereas the r-sAPPα may be contaminated by 23% h-sAPPα (Figure 2C). One μl of mouse CSF was sufficient to detect r-sAPPα as well as h-sAPPα and detection was linear for serially diluted samples. This level of sensitivity may allow the levels of the human transgenic sAPPα to be followed through the progression of the disease, which has never been reported previously.

**Table 5:** Measurement of sAPPα in mouse CSF

**Mice**	**r-sAPPα (ng/ml)**	**h-sAPPα (ng/ml)**
Wild-type (5)	137 ± 17	0
3xTg AD (7)	105 ± 8^#^	34 ± 6

CSF samples (2–5 μl) were collected from wild type or 3xTg AD mice and 1/5 or 1/10 dilutions of the CSF were tested in the r-sAPPα and in the h- sAPPα assays.^#^: This value includes 23% of h- sAPPα (see Figure 2C).

### sAPPα assay development: *in vivo* applications in human

**Figure 6:**
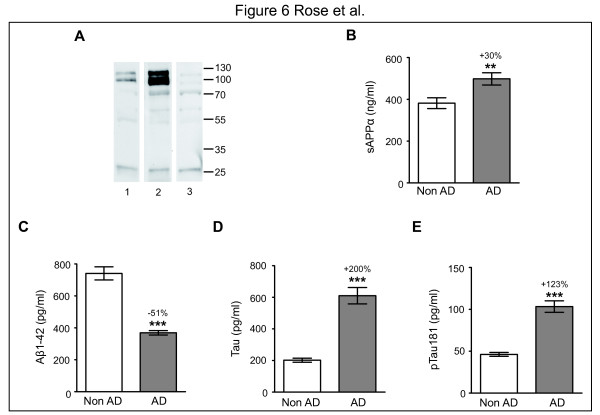
**Concentrations of different biomarkers in the CSF of two groups of patients previously diagnosed as AD and non-AD.A**: 4 μg of CSF proteins were separated in 10% SDS-PAGE for western blotting. h-sAPPα (two isoforms) present in CSF are recognized by both the 6E10 (1) and the 22C11 (2) antibodies. The anti-mouse secondary antibody (3) recognized non specifically some bands. **B**-**E**: Concentrations of biomarkers in the CSF of AD patients group were compared to those in the CSF of non-AD patients. **: p < 0.01; ***: p < 0.001.

We then assessed h-sAPPα levels in the CSF of AD and non-AD patients. High levels of h-sAPPα in the CSF determined using a multiplex technology were reported to be associated with AD [17,18,25]. In this study, we evaluated our h-sAPPα assay using CSF samples from patients who had been classified according to validated criteria as either non-AD or AD by a team specialized in the diagnosis of neurodegenerative disorders (CP, JD, JH). These patients were matched according to sex and age and significantly differ by the MMSE test (Table 6). We checked by western blotting in some CSF samples the presence of two sAPPα isoforms recognized by both 22C11 and 6E10 antibodies and the absence of specific detectable cleaved isoforms (Figure 6A). The two sAPPα isoforms of molecular weights (MW) 100 and 125 kDa detected are similar to that observed in soluble fraction of mouse brains (data not shown) and correspond to mature glycosylated forms of sAPPα 695 [27,39]. However we cannot exclude that the higher isoform correspond to the sAPPα 751 containing KPI domain and present in glial cells [37,39]. The bands of lower MW and lower intensity detected were non specific as recognized by the anti-mouse secondary antibody (Figure 6A). In our population, Aβ1-42 concentrations were lower (−51%; p < 0.001; Figure 6C) and Tau and pTau181 concentrations were higher (+200% and +123%, respectively; p < 0.001; Figure 6D, E) in the AD group than in the non-AD group. We found significantly higher sAPPα concentrations in AD CSF compared to non-AD CSF (+30%; p < 0.01; Figure 6B), which agrees with recent studies in which the sAPPα concentrations in CSF were determined by a multiplex technology [17,18,25].

**Table 6:** Patient characteristics

**Group (n)**	**Age ± SEM**	**M + F**	**MMSE ± SEM**
Non AD (28)	68.9 ± 2.0	14 + 14	23.5 ± 0.7
AD (32)	72.4 ± 1.6	12 + 19	19.3 ± 1.0 **

AD: patients with Alzheimer’s disease; non-AD: patients with other dementias; M: male; F: female. MMSE: mini mental state examination. **: p < 0.01.

Furthermore, our test detected h-sAPPα in as little as 0.5 μl of CSF, which is a vast improvement over ELISA tests and multiplex technology, which require sample volumes of 100 μl and 25 μl, respectively. The low amounts of CSF required by our test will allow large clinical and longitudinal investigations in patients to validate sAPPα as an AD biomarker.

## Conclusions

This is the first description of two highly sensitive tests that can be used to detect sAPPα in a variety of samples. Samples include the conditioned media of human neuroblastoma cells and mouse primary neurons, as well as very small samples of biological fluids, such as mouse and human CSF.

These new tools will have very wide applications from pre-clinical to clinical studies. Together, their rapidity, sensitivity and small incubation volumes will facilitate high-throughput investigations of drugs capable of increasing the secretion of endogenous sAPPα from primary neurons. The tests may be subsequently validated for *in vivo* analyses by using them to evaluate at different time points sAPPα levels in the CSF of wild-type mice or AD mouse models treated with molecules that exhibit activity in *in vitro* screening assays. Finally, the human sAPPα test will allow sAPPα levels to be measured in the CSF of patients participating in large clinical investigations.

## Methods

### sAPPα assays

The quantification of human and rodent sAPPα was achieved by adapting the HTRF technology developed by Cisbio international company (Bagnol ssur Cèze, France). It is a single step immunoassay that employs two primary antibodies. The first antibody for both tests is directed to an epitope in the N-terminal domain of the APP protein, which is common to humans and rodents (22C11; MAB 348 from Chemicon, Billerica, MA, USA), and coupled to Europium cryptate, a donor fluorophore. The second antibody in each assay is specific for the C-terminal domain of either h-sAPPα (6E10) or r-sAPPα (SIG-39153; both antibodies are from Signet laboratories, and purchased from Covance, Dedham, MA, USA). Both of the secondaries antibodies were coupled to an acceptor fluorophore, such as D2 (Figure 1). When the donor and acceptor fluorophores are brought together by a biomolecular interaction, i.e., when sAPPα is present in the sample, a portion of the energy captured by the donor fluorophore during excitation is released through a fluorescence emission at 620 nm, while the remaining energy is transferred to the acceptor. This energy is then released by the acceptor as a specific fluorescence at 665 nm and is proportional to the concentration of sAPPα in the sample.

Recombinant h-sAPPα, r-sAPPα and h-sAPPβ were produced in bacteria using a p-Gex vector and were purified from bacterial lysates using the glutathione-S-transferase system (GE Healthcare, Uppsala, Sweden), as previously described [40]. Their purity was checked by 10% SDS-PAGE (Bio-Rad, Hercules, CA, USA). The Aβ1-42 peptide was purchased from Bachem (Weil am Rhein, Germany). Fluorophore labeling of antibodies was done by Cisbio.

The assay was performed in a final volume of 20 μl in a 384-well white plate (Proxiplate; Perkin Elmer, Waltham, MA, USA). 5 μl of conditioned medium from SH-SY5Y neuroblastoma cell cultures or from mouse neurons in primary culture, 5 μl of rodent CSF dilutions (1/2.5, 1/5, 1/10), 5 μl of human CSF dilutions (1/2.5, 1/5, 1/10, 1/20), or a calibration curve (human or rodent recombinants APPα, at concentrations of 3–200 ng/ml) in a 50 mM phosphate buffer pH 7.0 containing 0.2% BSA and 10 mM DTT, were added to 5 ng of the cryptate conjugate antibody and 20 ng of the D2 conjugate antibody. Both antibodies were diluted in 50 mM phosphate buffer pH 7.0 containing 0.4 M potassium fluorure. All measurements were performed in duplicate. For the negative control, phosphate buffer replaced the calibration curve. The plate covered with a plate sealer was centrifuged for 1 min at 1000 x *g* and incubated overnight at room temperature. Then, the plate sealer was removed, and the plate was read on a compatible HTRF reader (Envision; Perkin Elmer, Waltham, MA, USA) at 620 and 665 nm. Results were calculated from the 665 nm/620 nm ratio and expressed in delta F: Delta F = ([test sample or calibration sample 665 nm/620 nm ratio − negative control 665 nm/620 nm ratio]/[negative control 665 nm/620 nm ratio])×100. Delta F is proportional to the sAPPα concentration.

### Cell cultures

SH-SY5Y human neuroblastoma cells were obtained from the ATCC and maintained at 37°C under 5% carbon dioxide/95% humidified air incubator in a DMEM medium, supplemented with Glutamax, 10% fetal calf serum (FCS), 0.1% penicillin/streptomycin. Cells were plated (3 × 10^4^ cells per well) in 96-well plates coated with 10 μg/ml collagen. The next day, when cells were 60–80% confluent, the conditioned medium was discarded and replaced by the same medium containing 1% N2 (Gibco, Paisley, UK) in place of FCS. Then, 10 μM GF-109203 (Sigma, St. Louis, MO, USA) and 30 μM TAPI 0 (Calbiochem, San Diego, CA, USA) were added 1 hour before adding 0.3 μM PDBu (Sigma, St. Louis, MO, USA) and the cells were incubated for 24 hours. After this period, conditioned media were collected, centrifuged at 800 ×*g* for 5 min at room temperature to remove any dead cells and the supernatant was immediately supplemented with 1X complete protease inhibitor cocktail (Roche, Bâle, Switzerland) and 5 μl duplicate aliquots were tested in the h-sAPPα assay or were stored at −20°C until the h-sAPPα assay.

Cortical neuron primary cultures were obtained from E16 Swiss mouse embryos. Neurons were grown in a defined medium free of serum and supplemented with hormones, proteins, and salts as previously described [41]. Briefly, dissociated cells were plated (5 × 10^4^ neurons per well) in 96-well plates coated with 1.5 μg/ml polyornithin. After six days *in vitro*, 60% of the conditioned medium was discarded and replaced by fresh medium. Then, 30 μM EGCG or 1 μM PACAP-27 (Sigma, St. Louis, MO, USA) were added for a 24 hours incubation. At the end of this period, conditioned media were collected, centrifuged at 800 × *g* for 5 min at room temperature and the supernatant was immediately supplemented with 1X complete protease inhibitor cocktail and 5 μl duplicate aliquots were tested in the r-sAPPα assay or were stored at −20°C until the r-sAPPα assay.

### Patients

60 patients were recruited from the Clinical and Research Memory Center (CMRR) in Paris Lariboisière Hospital, which is specialized in the care management of patients with cognitive disorders. We included in this study AD and non-AD patients for whom a lumbar puncture (LP) had been performed between November 1^st^, 2008 and November 1^st^, 2011 to investigate potential cognitive disorders. All patients had an extensive examination including clinical and neuropsychological evaluations, biological measurements and brain imaging. Based on all available information, patients were classified into two groups (32 AD and 28 non-AD). Complex or unclear cases were discussed, and diagnoses were then set by a multidisciplinary team of neurologists, geriatricians and neuropsychologists. AD patients fit the criteria for probable AD, as defined by the NINCDS-ADRDA [42], and had biochemical CSF results associated with AD (low Aβ1-42 levels, and high total Tau and pTau181 levels) [3-6].

Non-AD patients were diagnosed with other cognitive disorders, according to validated criteria, including: vascular dementia (n = 7), frontotemporal degeneration (n = 3), depression (n = 2), anxiety (n = 4), Lewy body disease (n = 2), intoxication (n = 1), supranuclear palsy (n = 1), chronic hydrocephalus (n = 2), alcoholism (n = 5), and sleep apnea syndrome (n = 1). Patients with unknown clinical diagnoses or who were not able to undergo testing or a magnetic resonance imaging were excluded. There was no Mild Cognitive Impairment patients in our study. LPs were performed on fasting patients in the month following their clinical diagnosis. CSF was collected in 12 ml polypropylene tubes (TC10PCS; CML, Nemours, France) in standardized conditions and transferred within 4 hours to the laboratory. It was then rapidly centrifuged at 1800 × *g* for 10 min at 4°C. A small amount of CSF was used for routine analysis, including total cell count, bacteriologic exam, total protein and glucose concentration. CSF was aliquoted in polypropylene tubes of 1 ml and stored at −80°C until use for further analysis.

Measurements of Aβ1-42, Tau and pTau181 levels in CSF were performed by sandwich ELISA according to the manufacturer’s protocols (Innogenetics, Ghent, Belgium). A neurobiological AD profile was established for all the patients in this study.

### SDS-PAGE and western blotting analysis

Human and rodent sAPPα recombinant proteins (0.5 μg), were separated in 10% SDS-PAGE (Criterion; Bio-Rad, Hercules, CA, USA), and stained with SimplyBlueSafeStain (Invitrogen Life Technologies, Paisley, UK).

For western blotting analysis, 0.6 ml of conditioned medium at six days *in vitro* from 4 × 10^5^ cortical neurons primary cultures were centrifuged at 800 x *g* for 5 min at room temperature. The supernatant was precipitated overnight at −20°C with five volumes of a cold mixture 10% trichloracetic acid/90% acetone. After centrifugation at 15,000 × *g* for 20 min at 4°C, the pellet was washed with cold acetone and dried. Then, the pellet was resuspended in Laemmli buffer and an equal volume was loaded in two wells in 10% SDS-PAGE. After migration, the proteins were transferred onto polyvinylidene fluoride (PVDF) membranes (Immobilon; Millipore, Billerica, MA, USA) and blocked in 5% low fat milk in Tris buffer saline, 0.02% Tween 20 (TBST). Membranes were incubated overnight with the appropriate primary antibody, 22C11 (1 μg/ml) or the rodent specific SIG-39153 (2 μg/ml) in TBST. Then after 5 washings in TBST, species-specific peroxidase-conjugated secondary antibodies were incubated 45 min at room temperature. After 5 washings the peroxidase signal was visualized using ECL (Western Blotting Detection Reagents; GE Healthcare, Uppsala, Sweden).

Human cerebrospinal fluid (4 μg protein) was diluted in Laemmli buffer and loaded in 10% SDS-PAGE. After migration, and transfer onto PVDF membranes, the membranes were processed as above with the appropriate primary antibody, 22C11 (1 μg/ml) or human specific 6E10 (2 μg/ml).

### Ethics statement

All animal procedures followed the French and European Union regulations. The protocols of animal anesthesia and biological fluid punctures were performed according to French government ethical laws decree 86/109 and approved by the local Ethics Committee (Direction départementale des services vétérinaires de Paris, service de la protection et santé animales et de la protection de l’environnement), permit number: 75–354.

For the clinical study, the research project was approved by the Ethics Committee of Paris University Hospitals (CEERB Bichat University Hospital, Paris, France). All patients or caregivers gave their written informed consent for CSF assessment.

### Mouse CSF sampling

We used seven triple transgenic mice (3xTg AD, 12-months-old) harboring three mutant genes: β-amyloid precursor protein (APPswe), presenilin-1 (M146V) knock-in, and Tau (P301L) [38] and five non-transgenic controls (14-months-old). CSF from anesthetized mice was taken from the cisterna magna using a glass capillary, according to the method of Fisher et al. [43]. The procedure was performed to keep blood contamination to a minimum. Samples of 2 to 5 μl were transferred directly to a microvial, centrifuged, and the supernatant immediately frozen on dry ice, and then stored at −20°C until rodent and human sAPPα assays were performed.

### Statistical analysis

Statistical differences between groups were analyzed using the unpaired Student’s *t*-test. Differences were considered significant if p < 0.05 (GraphPad Prism software, La Jolla, CA, USA).

## Abbreviations

Aβ1-42, β-amyloid peptide 1–42; AD, Alzheimer’s disease; BSA, bovine serum albumin; CMRR, Clinical and Research Memory Center; CSF, Cerebrospinal fluid; CV, Coefficient of variation; DTT, Dithiothreitol; EGCG, Epigallocatechin-3-gallate; ELISA, Enzyme-linked immunosorbent assay; h-sAPPα, Human sAPPα; h-sAPPβ, Human sAPPβ; MMSE, Mini-mental state examination; PACAP-27, Pituitary adenylatecyclase-activating polypeptide (27aa); MW, Molecular weight; PDBu, Phorbol 12,13 dibutyrate; PKC, Protein kinase C; pTau 181, Tau phosphorylated on threonine 181; PVDF, Polyvinylidene fluoride; TBST, Tris buffer saline; r-sAPPα, Rodent sAPPα; sAPPα, Soluble amyloid precursor protein alpha; sAPPβ, Soluble amyloid precursor protein beta; SEM, Standard error of the mean.

## References

[B1] Duyckaerts C Delatour B Potier MC Classification and basic pathology of Alzheimer disease Acta Neuropathol 2009 118 1 5 36 19381658 10.1007/s00401-009-0532-1

[B2] McKhann GM Knopman DS Chertkow H Hyman BT Jack CR Kawas CH Klunk WE Koroshetz WJ Manly JJ Mayeux R The diagnosis of dementia due to Alzheimer's disease: recommendations from the National Institute on Aging-Alzheimer's Association workgroups on diagnostic guidelines for Alzheimer's disease Alzheimer Dement 2011 7 3 263 269 10.1016/j.jalz.2011.03.005 PMC3312024 21514250

[B3] Blennow K Hampel H CSF markers for incipient Alzheimer's disease Lancet Neurol 2003 2 10 605 613 14505582 10.1016/s1474-4422(03)00530-1

[B4] Ibach B Binder H Dragon M Poljansky S Haen E Schmitz E Koch H Putzhammer A Kluenemann H Wieland W Cerebrospinal fluid tau and beta-amyloid in Alzheimer patients, disease controls and an age-matched random sample Neurobiol Aging 2006 27 9 1202 1211 16085339 10.1016/j.neurobiolaging.2005.06.005

[B5] Blennow K Hampel H Weiner M Zetterberg H Cerebrospinal Fluid and Plasma Biomarkers in Alzheimer Disease Nat Rev Neurol 2010 6 131 144 20157306 10.1038/nrneurol.2010.4

[B6] Mattsson N Rosen E Hansson O Andreasen N Parnetti L Jonsson M Herukka SK van der Flier WM Blankenstein MA Ewers M Age and diagnostic performance of Alzheimer disease CSF biomarkers Neurology 2012 78 7 468 476 22302554 10.1212/WNL.0b013e3182477eed PMC3280049

[B7] Esch FS Keim PS Beattie EC Blacher RW Culwell AR Olterstof T McClure D Ward PJ Cleavage of Amyloid beta peptide during constitutive processing of its precursor Science 1992 248 4959 1122 1124 2111583 10.1126/science.2111583

[B8] Anderson JP Esch DS Keim PS Sambamurti K Lieberburg I Robakis NK Exact cleavage site of Alzheimer amyloid precursor in neuronal PC-12 Neurosci Lett 1991 128 1 126 128 1922940 10.1016/0304-3940(91)90775-o

[B9] Wang R Meschia JF Cotter RJ Sisodia SS Secretion of the beta/A4 Amyloid Precursor Protein J Biol Chem 1991 266 25 16960 16964 1909332

[B10] Flammang B Pardossi-Piquard R Sevalle J Debayle D Dabert-Gay AS Thevenet A Lauritzen I Checler F Evidence that the amyloid-beta protein precursor intracellular domain, AICD, derives from beta-secretase-generated C-terminal fragment J Alzheimer Dis 2012 30 1 145 153 10.3233/JAD-2012-112186 22406447

[B11] Portelius E Price E Brinkmalm G Stiteler M Olsson M Persson R Westman-Brinkmalm A Zetterberg H Simon AJ Blennow K A novel pathway for amyloid precursor protein processing Neurobiol Aging 2011 32 6 1090 1098 19604603 10.1016/j.neurobiolaging.2009.06.002

[B12] Tian Y Crump CJ Li YM Dual role of alpha-secretase cleavage in the regulation of gamma-secretase activity for amyloid production J Biol Chem 2010 285 42 32549 32556 20675367 10.1074/jbc.M110.128439 PMC2952257

[B13] Mattson MP Cellular actions of beta-amyloid precursor protein and its soluble and fibrillogenic derivatives Physiol Rev 1997 77 4 1081 1132 9354812 10.1152/physrev.1997.77.4.1081

[B14] Chasseigneaux S Allinquant B Functions of Abeta, sAPPalpha and sAPPbeta: similarities and differences J Neurochem 2012 120 Suppl 1 99 108 22150401 10.1111/j.1471-4159.2011.07584.x

[B15] Caille I Allinquant B Dupont E Bouillot C Langer A Müller U Prochiantz A Soluble form of amyloid precursor protein regulates proliferation of progenitors in the adult subventricular zone Development 2004 131 9 2173 2181 15073156 10.1242/dev.01103

[B16] Taylor CJ Ireland DR Ballagh I Bourne K Marechal NM Turner PR Bilkey DK Tate WP Abraham WC Endogenous secreted amyloid precursor protein-alpha regulates hippocampal NMDA receptor function, long-term potentiation and spatial memory Neurobiol Dis 2008 31 2 250 260 18585048 10.1016/j.nbd.2008.04.011

[B17] Lewczuk P Kamrowski-Kruck H Peters O Heuser I Jessen F Popp J Burger K Hampel H Frolich L Wolf S Soluble amyloid precursor proteins in the cerebrospinal fluid as novel potential biomarkers of Alzheimer's disease: a multicenter study Mol Psychiatr 2010 15 2 138 145 10.1038/mp.2008.84 18663368

[B18] Lewczuk P Popp J Lelental N Kolsch H Maier W Kornhuber J Jessen F Cerebrospinal fluid soluble amyloid-beta protein precursor as a potential novel biomarkers of Alzheimer's disease J Alzheimer Dis 2012 28 1 119 125 10.3233/JAD-2011-110857 21971403

[B19] Lannfelt L Basun H Wahlund LO Rowe BA Wagner SL Decreased alpha-secretase-cleaved amyloid precursor protein as a diagnostic marker for Alzheimer's disease Nat Med 1995 1 8 829 832 7585189 10.1038/nm0895-829

[B20] Almkvist O Basun H Wagner SL Rowe BA Wahlund LO Lannfelt L Cerebrospinal fluid levels of alpha-secretase-cleaved soluble amyloid precursor protein mirror cognition in a Swedish family with Alzheimer disease and a gene mutation Arch Neurol 1997 54 5 641 644 9152122 10.1001/archneur.1997.00550170111022

[B21] Sennvik K Fastbom J Blomberg M Wahlund LO Winblad B Benedikz E Levels of alpha- and beta-secretase cleaved amyloid precursor protein in the cerebrospinal fluid of Alzheimer's disease patients Neurosci Lett 2000 278 3 169 172 10653020 10.1016/s0304-3940(99)00929-5

[B22] Colciaghi F Borroni B Pastorino L Marcello E Zimmermann M Cattabeni F Padovani A Di Luca M [alpha]-Secretase ADAM10 as well as [alpha]APPs is reduced in platelets and CSF of Alzheimer disease patients Mol Med 2002 8 2 67 74 12080182 PMC2039975

[B23] Olsson A Hoglund K Sjogren M Andreasen N Minthon L Lannfelt L Buerger K Moller HJ Hampel H Davidsson P Measurement of alpha- and beta-secretase cleaved amyloid precursor protein in cerebrospinal fluid from Alzheimer patients Exp Neurol 2003 183 1 74 80 12957490 10.1016/s0014-4886(03)00027-x

[B24] Perneczky R Tsolakidou A Arnold A Diehl-Schmid J Grimmer T Forstl H Kurz A Alexopoulos P CSF soluble amyloid precursor proteins in the diagnosis of incipient Alzheimer disease Neurology 2011 77 1 35 38 21700579 10.1212/WNL.0b013e318221ad47

[B25] Gabelle A Roche S Geny C Bennys K Labauge P Tholance Y Quadrio I Tiers L Gor B Chaulet C Correlations between soluble alpha/beta forms of amyloid precursor protein and Abeta38, 40, and 42 in human cerebrospinal fluid Brain Res 2010 1357 175 183 20713025 10.1016/j.brainres.2010.08.022

[B26] Szekeres PG Leong K Day TA Kingston AE Karran EH Development of homogeneous 384-well high-throughput screening assays for Abeta1-40 and Abeta1-42 using AlphaScreen technology J Biomol Screen 2008 13 2 101 111 18216395 10.1177/1087057107312778

[B27] De Strooper B Simons M Multhaup G Van Leuven F Beyreuther K Dotti CG Production of intracellular amyloid-containing fragments in hippocampal neurons expressing human amyloid precursor protein and protection against amyloidogenesis by subtle amino acid substitutions in the rodent sequence EMBO J 1995 14 20 4932 4938 7588622 10.1002/j.1460-2075.1995.tb00176.x PMC394596

[B28] Perret-Liaudet A Pelpel M Tholance Y Dumont B Vanderstichele H Zorzi W Elmoualij B Schraen S Moreaud O Gabelle A Cerebrospinal Fluid Collection Tubes: A Critical Issue for Alzheimer Disease Diagnosis Clin Chem 2012 58 4 787 789 22322825 10.1373/clinchem.2011.178368

[B29] Pica-Mendez AM Tanen M Dallob A Tanaka W Laterza OF Nonspecific binding of Abeta42 to polypropylene tubes and the effect of Tween-20 Clin Chim Acta 2010 411 21–22 1833 20655889 10.1016/j.cca.2010.07.019

[B30] Paquet C Latour F Saulnier I Hanon O [Multicenter study on lumbar puncture indication, clinical practice and feasibility] Rev Neurol Paris 2012 168 1 28 32 22153704 10.1016/j.neurol.2011.08.012

[B31] Hung AY Haass C Nitsch RM Qiu WQ Citron M Wurtman RJ Growdon JH Selkoe DJ Activation of protein kinase C inhibits cellular production of the amyloid beta-protein J Biol Chem 1993 268 31 22959 22962 8226807

[B32] Buxbaum JD Liu KN Luo Y Slack JL Stocking KL Peschon JJ Johnson RS Castner BJ Cerretti DP Black RA Evidence that tumor necrosis factor alpha converting enzyme is involved in regulated alpha-secretase cleavage of the Alzheimer amyloid protein precursor J Biol Chem 1998 273 43 27765 27767 9774383 10.1074/jbc.273.43.27765

[B33] Postina R Activation of alpha-secretase cleavage J Neurochem 2012 120 Suppl 1 46 54 21883223 10.1111/j.1471-4159.2011.07459.x

[B34] Kojro E Postina R Buro C Meiringer C Gehrig-Burger K Fahrenholz F The neuropeptide PACAP promotes the alpha-secretase pathway for processing the Alzheimer amyloid precursor protein FASEB J 2006 20 3 512 514 16401644 10.1096/fj.05-4812fje

[B35] Levites Y Amit T Mandel S Youdim MB Neuroprotection and neurorescue against Abeta toxicity and PKC-dependent release of nonamyloidogenic soluble precursor protein by green tea polyphenol (−)-epigallocatechin-3-gallate FASEB J 2003 17 8 952 954 12670874 10.1096/fj.02-0881fje

[B36] Obregon DF Rezai-Zadeh K Bai Y Sun N Hou H Ehrhart J Zeng J Mori T Arendash GW Shytle D ADAM10 activation is required for green tea (−)-epigallocatechin-3-gallate-induced alpha-secretase cleavage of amyloid precursor protein J Biol Chem 2006 281 24 16419 16427 16624814 10.1074/jbc.M600617200

[B37] Caporaso GL Gandy SE Buxbaum JD Ramabhadran TV Greengard P Protein phosphorylation regulates secretion of Alzheimer beta/A4 amyloid precursor protein Proc Natl Acad Sci U S A 1992 89 7 3055 3059 1557413 10.1073/pnas.89.7.3055 PMC48802

[B38] Oddo S Caccamo A Shepherd JD Murphy MP Golde TE Kayed R Metherate R Mattson MP Akbari Y LaFerla FM Triple-transgenic model of Alzheimer's disease with plaques and tangles: intracellular Abeta and synaptic dysfunction Neuron 2003 39 3 409 421 12895417 10.1016/s0896-6273(03)00434-3

[B39] Simons M de Strooper B Multhaup G Tienari PJ Dotti CG Beyreuther K Amyloidogenic processing of the human amyloid precursor protein in primary cultures of rat hippocampal neurons J Neurosci 1996 16 3 899 908 8558258 10.1523/JNEUROSCI.16-03-00899.1996 PMC6578819

[B40] Chasseigneaux S Dinc L Rose C Chabret C Coulpier F Topilko P Mauger G Allinquant B Secreted amyloid precursor protein beta and secreted amyloid precursor protein alpha induce axon outgrowth in vitro through Egr1 signaling pathway PLoS One 2011 6 1 e16301 21298006 10.1371/journal.pone.0016301 PMC3029320

[B41] Lafont F Rouget M Rousselet A Valenza C Prochiantz A Specific responses of axons and dendrites to cytoskeleton perturbations: an in vitro study J Cell Sci 1993 104 Pt2 433 443 8505371 10.1242/jcs.104.2.433

[B42] McKhann G Drachman D Folstein M Katzman R Price D Stadlan EM Clinical diagnosis of Alzheimer's disease: report of the NINCDS-ADRDA Work Group under the auspices of Department of Health and Human Services Task Force on Alzheimer's Disease Neurology 1984 34 7 939 944 6610841 10.1212/wnl.34.7.939

[B43] Fischer DF Hol EM Hobo B Van Leeuwen FW Alzheimer-associated APP+1 transgenic mice: Frameshift β-amyloid precursor protein is secreted in cerebrospinal fluid without inducing neuropathology Neurobiol Aging 2006 27 10 1445 1450 16214267 10.1016/j.neurobiolaging.2005.09.010

